# An evaluation of osteopathic treatment on psychological outcomes with patients suffering from chronic pain: A prospective observational cohort study collected through a health and well-being academy

**DOI:** 10.1177/2055102918774684

**Published:** 2018-05-10

**Authors:** Darren J Edwards, Craig Toutt

**Affiliations:** Swansea University, UK

**Keywords:** acceptance and commitment therapy, anxiety, chronic pain, depression, mental health, osteopathy

## Abstract

Co-morbid mental health conditions such as anxiety, depression and fear avoidance are often associated with chronic pain. This novel study aimed to explore the impact of osteopathic treatment on several psychological outcome measures relating to anxiety, depression, mental health and fear avoidance for a chronic pain population receiving osteopathic treatment over a 2-week period. The findings show that there were significant reductions in anxiety, pain, mental health dysfunction and improvements in self-care. These results are promising, and it is suggested that now a full-scale randomised controlled trial should be conducted.

## Introduction

One of the leading forms of pain and disability globally is low back pain (LBP; Freburger et al., 2009), where the majority of the population experience this at some point in their lives (Klyne et al., 2017; Yang et al., 2016). It leads to the greatest frequency of pharmacological prescriptions, medical claims and recorded authorised leave from work in the world (Driscoll et al., 2014). In terms of annual cost, it is estimated that in the United Kingdom alone, just for LBP, 116 million days of work are lost, 1 million hospital appointments and 5 million general practitioner (GP) visits are made^1^ (Briggs et al., 2009).

In addition to the impact on economic cost and physical disability, chronic pain can have an impact on psychological outcomes in the form of co-morbid mental health conditions. This can occur in about 35 per cent of cases where depression, anxiety and social isolation are present (Miller and Cano, 2009). Chronic pain’s relation with anxiety and depression is thought to be associated with psychological inflexibility as a result of experiential avoidance (McCracken et al., 2004, 2007; McCracken and Samuel, 2007; McCracken and Yang, 2006).

One form of treatment for chronic pain, suggested by the recent guidelines by National Institute for Health and Care Excellence (NICE; Bernstein et al., 2017), is to use manual therapy as part of a broader package of treatments (e.g. in conjunction with exercise therapy and psychological treatment) to help reduce LBP and co-morbid mental health conditions. One type of manual therapy called osteopathy (osteopathic manipulative therapy; OMT) has been supported in its effectiveness to reduce chronic pain in several areas, such as chronic neck pain, as highlighted in a Cochrane review which identified 33 relevant clinical trials (Gross et al., 2004). More specific studies have directly compared osteopathy with standard primary care, for example, the UK BEAM trial team demonstrated that OMT followed by exercise was more effective than standard primary care at 3 and 12 months for persistent LBP in reducing disability (UK BEAM trial team, 2004). In addition to this, a previous study published in *The New England Journal of Medicine* demonstrated that OMT was useful for reducing subacute LBP (Andersson et al., 1999). Accordingly, a UK report which explored 49 systematic reviews concluded that spinal mobilisation and manipulation was effective for acute, subacute and chronic LBP (Bronfort et al., 2010).

It is perhaps a shame that not more studies have focused on the effect of OMT in reducing the co-morbid psychological disorders of pain, such as depression and anxiety as demonstrated by a recent systematic review on the psychosocial impact of osteopathy and manual therapy (Saracutu et al., 2018). In this review, only four randomised controlled trials (RCTs) were assessed as having high quality when using the Critical Skills Appraisal Programme (CASP).

These included an RCT (Bialosky et al., 2009) which found that for patients receiving spinal manipulative therapy, their state anxiety positively correlated to their changes in pain sensitivity; however, there was no mention that this therapy was delivered by an osteopath specifically. For depression, another RCT (Moustafa and Diab, 2015) found that after 1 hour, three times a week and for 12 weeks programme, there were significant differences between the experimental and control groups for Becks Depression Inventory (BDI) scores after a 1-year follow-up, but again, this was not specific to OMT. Licciardone et al. (2013) found that patients with comorbid depression did not respond favourably to OMT; however, the impact of OMT on psychological outcomes was not specifically explored. In another study, there were a significant interaction between trait anxiety and pain, but there were no significant effects of manual therapy on depression (Lopez-Lopez et al., 2015), in addition to this, there was again no mention that an osteopath delivered the intervention.

Outside of these strict methodologically orientated RCTs, Williams et al. (2003) conducted a pragmatic trial for spinal pain in primary care which was delivered by an osteopath and found that OMT improved the mental health score of the SF-12 measure. From this, Williams (2007) later suggested that perhaps the psychological benefits of spinal manipulation could be optimised by integrating cognitive behavioural principles (e.g. in the form of cognitive behavioural therapy (CBT)) into the routine of osteopathic practice. In addition to this, it has been identified (Westmoreland et al., 2007) through a qualitative interview that the psychological benefits of OMT included reassurance, improved understanding of the condition, removal of fear and a more positive mental approach to the condition with a focus on improvement.

Another study which was identified by the Saracutu et al. (2018) review and which was scored at medium quality is particularly relevant to this study because of the broad range of measures used. This RCT (Castro-Sanchez et al., 2011) had explored the impact of massage-myofascial release therapy effects on anxiety, depression and quality of life with patients with fibromyalgia; however, again, this was not specific to OMT. This study used the 36-item SF-36 to measure quality of life of functional state, emotional well-being and general health; BDI to measure depression; and the State Trait Anxiety Inventory (STAI) to measure both state and trait anxiety. The findings demonstrated a significant improvement in trait anxiety and quality-of-life factors but not depression immediately after the 20-week intervention and 1-month post intervention.

This Castro-Sanchez et al. study as well as other studies such as Williams et al. (2003) give some promise for finding similar positive psychological effects with OMT interventions more generally for back and neck pain. Therefore, this is the aim of this study, to investigate whether OMT can reduce pain, anxiety, depression, fear avoidance and mental health dysfunction and improve quality-of-life dimensions where we hypothesise that they will. In doing this, this study will investigate OMT delivered through a Health and Wellbeing Academy (HWBA) cohort, with patient referrals from multiple pathways at Swansea University.

## Methodology

### Participants

In this study, a modest sample size was chosen. A G*Power analysis (Faul et al., 2007) was conducted with power selected at 0.8, alpha at 0.05 and with an a priori moderate effect size of *d* = 0.5 $\left(\eta_{p}^{2}=0.06\right)$ selected (based on the criteria of Cohen (1988)). 0.8 power was selected because it is suggested that studies should not have more than a 20 per cent probability of making a type 2 error, where the hypothesis is rejected when it should not have been (Cohen, 1988). The a priori power calculation suggests that a one-way univariate repeated-measures analysis of variance (ANOVA) requires 28 participants. A larger sample was chosen to account for possible attrition where patients fail to complete the study. Therefore, a purposive sample of 74 patients were obtained through the multi-pathway HWBA at Swansea University which consisted of referrals from Abertawe Bro Morgannwg University (ABMU) Health board, the general public directly and local GPs. Patients were excluded if they had received OMT in the past and were only included if they had been in pain for 3 months, as defined as chronic pain (Lumley et al., 2011; see Table 1). Of those included 16 failed to complete the questionnaires, leaving a total of 58 completed questionnaires.

**Table 1.** Demographic data, age range, area of pain, anxiety and depression scores.

Total	Age ranges, mean (SD)	Areas of body affected by pain	Anxiety (HADS)	Depression (HADS)	Mental disorder (GHQ)
*N *= 58	38.40 (12.95)	Lower back = 39	Mild = 9	Mild = 7	None = 35
		Upper back = 15	Moderate = 4	Moderate = 4	Present = 23
		Mid back = 14	Severe = 6	Severe = 3	
			Total ratio = 1:3	Total ratio = 1:5	Total ratio = 1:3

SD: standard deviation; HADS: Hospital Anxiety and Depression Scale; GHQ: General Health Questionnaire.

*N* = number.

Note that for areas of body inflicted with pain, there can be multiple areas affected so the total count of these will be higher than the total patient sample. Also, the ratio reflects the number of individuals in the cohort (expressed as a ratio) as having a psychological disorder.

### Study design

This was a prospective observational cohort study, where outcome measures were taken at three points in time and immediate effects recorded. The cohort is defined as a multi-pathway Health and Wellbeing cohort obtained through Swansea University.

### Ethical approval

Ethics were approved through the University Research Ethics Council (REC) and Health Research Authority (HRA).

### Materials

A total of six questionnaires were provided to the patients which included the demographics questionnaire.

#### Demographic questionnaire

The demographic questionnaire asked about age, site of pain and length of time the patient had suffered with this pain.

#### EuroQol five dimensions

The EuroQol five dimensions (EQ5D) is a measure for health-related quality-of-life (HRQOL) statuses. It has five components which assess mobility, self-care, usual activities, pain and discomfort, as well as anxiety. It also has a visual analogue scale (VAS) for measuring current health status. Scores for these were calculated for each of these five subsections as well as including the VAS and total EQ5D score of all five subsections. The EQ5D has high validity, as it correlates well with other health-related questionnaires such as the SF-36 (*r* = 0.61, *p* < 0.0001) and Parkinson’s d﻿isease q﻿uestionnaire (PDQ-39) (*r* = −0.75, *p* < 0.0001; Schrag et al., 2000).

#### Short-form McGill Pain Questionnaire

This is a scale for rating the quality and intensity of patient pain. This has high test–retest reliability (0.45–0.70), as well as validity as it correlates well with the other pain questionnaires such as the Western Ontario and McMasters Universities Pain Scale (*r* = 0.36, *p* < 0.01; Hawker et al., 2011).

#### General Health Questionnaire 12

This is a psychometric screening tool to identify common psychiatric conditions. It assesses the patients’ current state mental health and asks if it is different to their usual state; therefore, it is sensitive to short-term psychiatric disorders. It has high internal consistency with a Cronbach’s alpha coefficient of 0.76 (Sanchez-Lopez Mdel and Dresch, 2008).

#### Hospital Anxiety and Depression Scale

This scale assesses both anxiety and depression scores. It has high concurrent validity as it correlates well with other questionnaires such as the BDI (Hospital Anxiety and Depression Scale–Anxiety (HADS-A), *r* = O.64; Hospital Anxiety and Depression Scale–Depression (HADS-D), *r* = 0.72). It also has high internal consistency with a Cronbach’s alpha coefficient of 0.6 (Bjelland et al., 2002).

#### Fear-Avoidance Beliefs Questionnaire

This measure was designed to explore fear and avoidance behaviour, specifically focusing on patient beliefs about physical activity and work. It has a high internal consistency with a Cronbach’s alpha coefficient of 0.88 and 0.77 (Waddell et al., 1993).

### Procedure

Patients were recruited through posters placed in the reception areas, which clearly stated the inclusion and exclusion criteria. Following this, and in terms of screening for eligibility, once the participant contacted the researcher for further details of the study, they were again asked if they had received OMT in the past and how long their current pain had lasted. Form this, they were only included in the study if they had been in pain for 3 months and had not have received OMT prior to this present time.

In order to minimise any coercion, the patients interested in the study were free to contact the main researcher for further details and were ensured that participation was not compulsory as per the poster instructions. Once recruited, the patients were given the questionnaires in paper form during consultation in the following order: demographics questionnaire, EQ5D, Short-form McGill Pain Questionnaire (SF-MPQ), General Health Questionnaire (GHQ), HADS and FABQ.

They were given the same questionnaires at three different time intervals: baseline, midpoint and endpoint. Baseline was the starting point before any OMT had occurred, midpoint was the second week of treatment and endpoint was the third week of treatment. Some of the patients went on to receive further treatment; however, only a 2-week period was recorded for the purposes of this study, with no long-term follow-up. This limited time frame was selected because the director of the HWBA did not want to potentially distress the participants with a follow-up long-term set of questionnaires.

The osteopathic treatment for all patients was delivered by fourth-year (final year) osteopathic students as part of Swansea University’s Osteopathic Advanced Initial Degree programme leading to a professional qualification called Master in Osteopathy (M.Ost). The students were all supervised by qualified osteopaths. As part of this M.Ost programme, students utilise the HWBA to treat patients.

### Data analysis

A Shapiro–Wilk test was used to confirm that the data were normally distributed (*p* > 0.05), thus justifying the use of parametric tests. General linear models consisting of several one-way univariate repeated-measures ANOVA were used to analyse the differences between the independent variable (point in time); baseline, midpoint and endpoint, with the dependent variables being the questionnaire patient outcome scores.

Although a multivariate analysis of variance (MANOVA) could have incorporated all of the dependent variables (questionnaire outcome measures) in a single analysis, this was deemed inappropriate as Stevens (1992) suggests that this should be only done when the dependent variables (DVs) share some conceptual meaning and are linearly related in some way. Although some of these measures were psychological in nature, others related to pain and mobility. There was no reason to assume that these were sharing a strong conceptual meaning, or at all linearly related as they each accessed different aspects of psychology, mobility or pain.

In addition to this, a per-protocol analysis was conducted and not an intention-to-treat (ITT) analysis, which analysed only the participants who completed the protocol, and with no missing data, as this was an evaluation and not a full RCT, so assumptions about randomisation bias which compromise the internal validity of the results cannot be made (Del Re et al., 2013). The advantage of the per-protocol analysis is that because the estimate of treatment effects are conservative in an ITT analysis this makes it more prone to type two errors than a per-protocol analysis, so ITT analysis should only be used with RCTs in order to mitigate randomisation bias (Del Re et al., 2013).

## Results

### Demographic results

See Table 1 for the participant demographics of age, areas affected by pain, and severity of anxiety, depression and mental health.

### Descriptive results

Table 2 shows the mean and standard deviations for the scores of the outcome measures.

**Table 2.** Mean and standard deviation (SD) of the scores for each outcome measure over the three points in time.

Outcome measure	Baseline measure Mean (SD)	Midpoint measure Mean (SD)	Endpoint measure Mean (SD)	*F* value	*p* value	$\eta_{p}^{2}$
EQ5D Mobility	1.74 (0.890)	1.71 (0.701)	1.55 (0.776)	2.056	0.133	0.035
EQ5D Mobility	1.71 (0.701)	1.74 (0.890)	1.55 (0.776)	2.056	0.133	0.035
EQ5D Self-care	1.09 (0.339)	1.34 (0.715)	1.19 (0.576)	5.243	<0.01**	0.084
EQ5D Activities	2.45 (0.994)	2.19 (0.712)	2.02 (0.868)	6.411	<0.01**	0.101
EQ5D Pain	3.09 (0.708)	2.74 (0.715)	2.52 (0.822)	14.367	<0.001***	0.201
EQ5D Anxiety	1.74 (1.001)	1.64 (1.021)	1.48 (0.922)	5.085	<0.01**	0.149
EQ5D VAS	69.07 (18.441)	68.78 (22.777)	71.71 (22.029)	0.514	0.600	0.009
EQ5D Total	77.12 (21.268)	80.05 (25.830)	80.40 (21.895)	0.487	0.616	0.008
McGill Sensory	6.86 (4.781)	7.29 (5.755)	6.78 (4.706)	0.490	0.614	0.009
McGill Affective	1.41 (2.169)	1.24 (1.931)	0.90 (1.398)	2.365	0.099	0.040
McGill Total	8.12 (5.354)	8.52 (7.385)	7.74 (5.581)	0.582	0.560	0.010
McGill VAS	5.078 (2.343)	4.509 (2.143)	3.651 (2.357)	10.572	<0.001***	0.156
GHQ12	3.47 (3.738)	2.62 (3.838)	2.02 (2.947)	9.130	<0.001***	0.138
HADS Anxiety	6.38 (4.920)	5.71 (4.542)	5.19 (4.861)	6.633	<0.01**	0.158
HADS Depression	4.74 (4.245)	4.47 (4.457)	4.33 (4.127)	0.635	0.522	0.022
HADS Total	11.12 (8.141)	10.38 (8.504)	9.52 (9.291)	4.050	<0.05*	0.066
Fear Physical	10.98 (6.010)	9.78 (5.275)	9.84 (5.001)	1.880	0.1575	0.032
Fear Work	9.21 (8.418)	8.43 (8.982)	8.41 (9.046)	0.418	0.660	0.007

EQ5D: EuroQol five dimensions; VAS: visual analogue scale; HADS: Hospital Anxiety and Depression Scale; GHQ12: General Health Questionnaire 12.

* *p* < 0.05, **p < 0.01, ***p < 0.001.

### Inferential statistics

Table 2 shows the outcomes of the series of one-way univariate ANOVAs which compared baseline, midpoint and endpoint for each of the questionnaires’ outcomes and their subsections. The following were significant (see Table 2): EQ5D Self-care increased; EQ5D Activities decreased; EQ5D Pain decreased; EQ5D Anxiety decreased; McGill VAS overall pain intensity decreased; GHQ12, current mental disorder decreased; HADS Anxiety decreased; HADS Total decreased.

There was no significant difference for the following: EQ5D mobility decreased; EQ5D VAS increased; EQ5D Total increased; McGill Sensory decreased; McGill Affective decreased; McGill Total decreased; HADS Depression decreased; Fear Physical decreased; Fear Work decreased.

## Discussion

This present service evaluation sought to identify whether OMT was effective at reducing co-morbid mental conditions such as anxiety, depression and fear avoidance. Previous studies have identified the usefulness of OMT in reducing pain and disability (Andersson et al., 1999; Gross et al., 2004; UK BEAM trial team, 2004); however, very few OMT studies to date have explored whether OMT reduces psychological co-morbid psychological conditions.

Overall, it was interesting to note that self-care increased while pain, anxiety and mental health disorder significantly decreased. This improvement in self-care is consistent with the qualitative findings of a previous study (Westmoreland et al., 2007) who found that there was an increase in positive self-improvement focus after OMT. The improvement in anxiety and quality-of-life measures is also consistent with a previous RCT which also found a significant reduction in anxiety and improvements in quality-of-life factors but no significant impact on depression (Castro-Sanchez et al., 2011). In addition to this, there were consistencies with other RCTs who found improvements in state anxiety (Bialosky et al., 2009; Moustafa and Diab, 2015) and trait anxiety (Lopez-Lopez et al., 2015) but again not depression (Moustafa and Diab, 2015).

These results are promising and demonstrate the effectiveness of OMT to reduce co-morbid psychological disorders, though should be considered with caution as this was an evaluation and without a control, which focused on just immediate effects. Due to these positive psychological effects, an RCT should follow which would include a waitlist control group, thus control for possible confounds such as time effects (i.e. reductions in psychological disorders due to the natural passage of time). Long-term effects should also be explored.

Although OMT was successful at reducing anxiety, mental dysfunction and pain, it was not successful at significantly reducing depression and fear avoidance (though these did reduce). Therefore, in the future, in addition to the OMT, a psychological intervention could be applied to reduce any depression and fear avoidance that a chronic pain population may have, such as through combining it with a low intensity third wave CBT called acceptance and commitment therapy (ACT). ACT has been shown to have a positive effect at reducing experiential avoidance so may be useful with reducing the fear avoidance and depression (which OMT did not significantly reduce) through promoting a mindful and cognitively flexible experience (Hayes et al., 1999; McCracken et al., 2007).

This is also what Williams (2007) suggested through a systematic review. It was suggested there that further optimising of the psychological effects of osteopathy through integrating cognitive behavioural principles could be made, whereby greater integration is made in standard osteopathic care. In addition to this, Pincus and McCracken (Pincus et al., 2013) agreed with this in principle, but highlight that in most cases these interventions have only shown moderate improvements at best and without long-term improvements. They, therefore, suggested that any additional psychological interventions applied with OMT should be targeted at certain clinical subgroups (high risk) and should be theoretically driven.

The most common forms of treatment for these types of disorders is CBT, which uses a symptom reduction approach through thought reconstruction, with a focus on removing or reducing the disorder symptoms (Beck, 2011). ACT is different to more traditional (second wave) therapies such as CBT as it emphasises psychological flexibility through increasing acceptance, mindfulness, diffusion, values and commitment skills which helps to alter the clients’ relation to her thoughts and experiences (Hayes et al., 2011; Strosahl and Wilson, 1999).

Recently, ACT has been applied to at least one osteopathic clinical trial with some success (Carnes et al., 2017) and another feasibility study with positive preliminary findings (Nanke and Abbey, 2017). There are, however, some problems with this combined approach, as it is difficult to know for sure whether it was the OMT or the ACT intervention which leads to the positive psychological effects. There are also possible adverse effects to this approach, where acceptability may be low, which may need to be explored too. So, perhaps, as Pincus et al. (2013) suggest this should be targeted at high-risk subgroups within this population and with greater theoretical consideration.

The main limitation of this study was that it was an evaluation study and not an RCT; therefore, it did not include a waitlist control group, so these results should be considered with caution. There was also no follow-up data obtained as the data were collected immediately at baseline, after 1 and 2 weeks. This follow-up assessment would be interesting to include in future work to increase confidence in these findings.

In conclusion, this study has demonstrated that OMT was effective at reducing pain, anxiety and psychiatric disorders, but not effective at reducing depression and fear avoidance. If this studies’ findings can be replicated and show significant differences in comparison to a control and over a longer period, then this will become very exciting for future work in this area. Interventions such as ACT have been particularly useful at reducing depression and fear avoidance; therefore, this may be useful to include as a psychological intervention alongside osteopathic treatment in standardised practice for some subgroups.

## Notes

1. This is the most recent general epidemiological study that we could find in relation to the impact on the UK National Health Service (NHS), GP time and impact on work days lost, and more recent studies of this kind are needed.
